# Stakeholders’ Perspectives for the Development of a Point-of-Care Diagnostics Curriculum in Rural Primary Clinics in South Africa—Nominal Group Technique

**DOI:** 10.3390/diagnostics10040195

**Published:** 2020-04-01

**Authors:** Nkosinothando Chamane, Desmond Kuupiel, Tivani Phosa Mashamba-Thompson

**Affiliations:** 1 Department of Public Health Medicine, School of Nursing and Public Health, University of KwaZulu-Natal, Durban 4041, South Africa; desmondkuupiel98@hotmail.com (D.K.); Mashamba-Thompson@ukzn.ac.za (T.P.M.-T.); 2 Department of Public Health, University of Limpopo, Polokwane 0723, South Africa

**Keywords:** quality HIV point-of-care-diagnostics, nominal group technique, stakeholder engagement

## Abstract

Poor knowledge and adherence to point-of-care (POC) HIV testing standards have been reported in rural KwaZulu-Natal (KZN), a high HIV prevalent setting. Improving compliance to HIV testing standards is critical, particularly during the gradual phasing out of lay counsellor providers and the shifting of HIV testing and counselling duties to professional nurses. The main objective of this study was to identify priority areas for development of POC diagnostics curriculum to improve competence and adherence to POC diagnostics quality standards for primary healthcare (PHC) nurses in rural South Africa. Method: PHC clinic stakeholders were invited to participate in a co-creation workshop. Participants were purposely sampled from each of the 11 KwaZulu-Natal Districts. Through the Nominal Group Technique (NGT), participants identified training related challenges concerning delivery of quality point of care diagnostics and ranked them from highest to lowest priority. An importance ranking score (scale 1–5) was calculated for each of the identified challenges. Results: Study participants included three PHC professional nurses, one TB professional nurse, one HIV lay councilor, one TB assistant and three POC diagnostics researchers, aged 23–50. Participants identified ten POC diagnostics related challenges. Amongst the highest ranked challenges were the following:absence of POC testing Curriculum for nurses, absence of training of staff on HIV testing and counselling as lay counsellor providers are gradually being phased out,. absence of Continuous Professional Development opportunities and lack of Staff involvement in POC Management programs. Conclusion: Key stakeholders perceived training of PHC nurses as the highest priority for the delivery of quality POC diagnostic testing at PHC level. We recommend continual collaboration among all POC diagnostics stakeholders in the development of an accessible curriculum to improve providers’ competence and ensure sustainable quality delivery of POC diagnostic services in rural PHC clinics.

## 1. Introduction

Point-of-care (POC) diagnostic testing is defined as timely clinical testing performed during patient consultation when the result will be used to take appropriate action, which will lead to an improved health outcome [1]. POC diagnostic testing plays a critical role in healthcare access in settings with limited laboratory infrastructure. Early diagnosis and rapid initiation of treatment is key to controlling infectious diseases including HIV/AIDS [1,2]. Improving the quality of diagnostic services is therefore essential for improved access to quality primary healthcare (PHC) services. In South Africa an HIV Counselling and Testing (HCT) campaign was first launched in April 2010. During this time in PHC clinics HIV/AIDS testing was done by lay counselors, who were permanently employed staff sent for training specific to performing HIV/AIDS rapid testing and counselling. Their role was solely to provide HIV testing and counselling services. In 2014 the South African Department of Health then announced the gradual phasing out of lay councilors and adding HIV testing duties to professional nurse consultation duties. This shift of duties may lead to added pressure on clinics who already suffer because of staff shortages. Moreover, poor knowledge and adherence to quality assurance as well as HIV testing standards remain a challenge especially in resource limited areas [3]. This is a concern because quality assurance programs, which include regular calibration of instruments, participation to external quality assessment schemes, adherence to standard operating procedures and test operator competency as well as running of internal quality control samples on every test day have been shown to ensure accuracy of POC diagnostic testing [4,5]. Factors contributing to this problem include poor access to laboratory infrastructure, training resources and institutions due to clinics being situated in deep rural areas, time constraints and lack of motivation to engage in new interventions [3,6,7].

Various initiatives have been introduced towards addressing this challenge. They include the Rapid HIV testing quality improvement initiative (RTQII) introduced to seven countries in 2013, including Tanzania, Ethiopia and Kenya [8]. The goal of RTQII was to scale up coverage of HIV rapid testing quality improvement (QI) and assurance activities as well as to improve the quality and safety of rapid testing services [8]. The RTQII findings emphasized provision of standard operating procedures (SOPs), onsite supervision, job aiders and orientation of providers on the importance of compliance to HIV rapid testing standards as the main tactics which led to quality improvement [9].

In this study, we involved PHC clinic-based POC diagnostics stakeholders in a co-creation workshop to identify priority areas for the development of point-of-care (POC) diagnostics curriculum to improve competence and adherence to POC diagnostics quality standards for PHC nurses in rural South Africa. The findings of this study have the potential to inform policy making concerning Continuous Professional Development (CPD) interventions aimed at improving the competency of PHC health workers on POC diagnostics services. This will further contribute towards healthcare systems strengthening, through improving and ensuring provision of quality, reliable and sustainable POC diagnostic services.

## 2. Materials and Methods

### 2.1. Study Design

The Nominal Group Technique (NGT) was employed to enable engagement with representative key stakeholders from 11 districts of KwaZulu Natal (KZN). We defined key stakeholders as PHC workers with experience in performing POC diagnostic services in rural KZN PHC clinics and researchers in the field of POC diagnostics. NGT is defined as a process to identify strategic problems and to develop appropriate and innovative interventions to address them [10]. The NGT processes is commonly applied to homogenous groups and it involves four main phases—(i) Nominal or silent phase, where participants individually consider their personal responses to a presented question and write them down; (ii) Item generation phase, where individual participants take turns to share their responses with the group. The items generated are recorded without being discussed; (iii) Discussion and clarification phase, where group members discuss and ask questions in order to clarify items on the list and elaborate on their responses. During this phase, items with similar meanings are combined and duplicate items can be removed; (iv) Voting phase, here each participant is asked to prioritize the listed items by assigning ranks to them. The ranking results are then collated to produce a single list of priorities for the wider group [11,12]. Application of this process in this study is discussed below.

#### 2.1.1. Study Participants

We invited key stakeholders of PHC-based POC diagnostics to participate in a Nominal group co-creation workshop. The NGT team comprised of three Professional nurses, one TB professional nurse, one TB assistant, one HIV/AIDS lay councilor, two experienced researchers, the primary researcher (as facilitator) and one research assistant. Detailed characteristics of participants are presented in the next section.

#### 2.1.2. Sampling Strategy

A purposeful sampling strategy was used to select representative clinics to participate in this study. This sampling technique involves identifying and selecting participants with practical knowledge and experience of PHC-based POC diagnostics. This study was conducted as a follow up to the cross-sectional survey of 100 randomly selected clinics in KZN rural PHC clinics [3,13]. The survey was aimed at demining the accessibility, availability and utility of POC diagnostic services in rural PHC clinics [13]. Clinics identified to have the highest availability and usage of POC diagnostics following the survey were selected to participate in this study. We thus included one PHC clinic from each of the 11 KZN districts with the highest availability and usage of POC tests. Clinics with low HIV POC diagnostics availability and usage were excluded due to minimal experience in HIV testing as reported in a previous audit [3].

#### 2.1.3. NGT Process

The PI (NC) facilitated the workshop with the help of a trained research assistant (PS). The four phase NGT was performed to achieve the objective of this study. Prior to the meeting of all key stakeholders, a pre-elicitation technique [14] was employed, where collaborators were sent an invitation together with a brief on the program of the day. The brief included the purpose to create a platform for key stakeholders to come together and determine training-related challenges affecting delivery of quality point of care diagnostics services in PHC clinics and to identify priority areas to be addressed in order to overcome the challenges. The main aim of the NGT was to bring together key stakeholders to identify priority areas for the development of a POC diagnostics curriculum.

**Figure 1:**
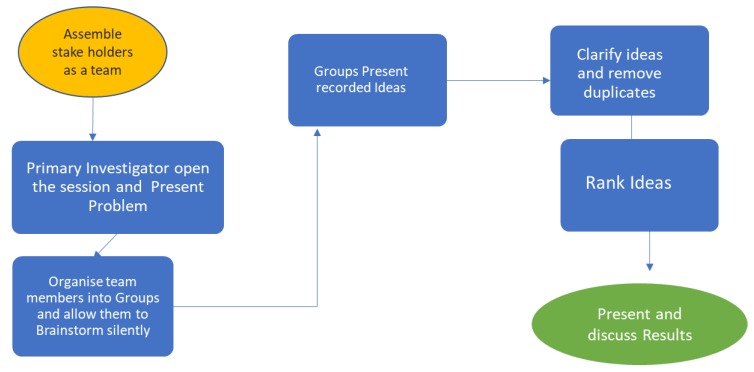
Nominal Group Technique (NGT) session to identify training related challenges with the delivery of quality point of care diagnostics.

At the opening of the session all participants were given an opportunity to introduce themselves, sharing their current positions as well as the number of years of experience in the field of HIV/AIDS testing. Following this, the PI (NC) provided a background to the workshop and presented the program of the day as illustrated in Figure 1. The participants were then separated into two sub-groups of four. The sub-groups were provided with a set of sticky notes, pens, markers and a flip chart sheet.

The PI (NC) posed the following question to participants, to start the workshop: what training related challenges do you encounter with regards to the delivery of quality HIV/AIDS Rapid tests? The following steps were followed to answer this question:

#### 2.1.4. Silent Brainstorming

Participants were given up to 10 min to consider the question and note down all the relevant ideas that came to mind. Discussions were prohibited during this period, however the participants could raise their hands for the attention of the facilitator if in need of clarity on the above question.

#### 2.1.5. Group Discussion

Group members were given another 10 min to share their ideas within their groups, group them into themes as they emerged and then stick them on the flip chart sheet to be presented to the whole workshop group.

#### 2.1.6. Group Presentations and Clarification

Each sub-group selected one representative to present their ideas according to the themes they had agreed upon. The facilitator encouraged questions and discussions during the discussion sessions. This process was also used as an opportunity to probe the presenters for further explanations as well as for the wider team to discuss and clarify presented ideas. During this process the research assistant collated all the ideas and together with the facilitator highlighted similar themes and removed duplicates. The collated results were presented to the wider group as priority areas to be ranked during the ranking session.

#### 2.1.7. Ranking of Ideas

The ranking process followed the strategy suggested by Delbecq et al. [15] of ranking ideas through assigning a value to an idea according to its priority. Ranking is usually preferred by many researchers, because scores can be quickly tallied and the results can be interpreted and discussed within the same session [16]. Participants were given a break, while the facilitator through the use of an online form software (Google^®^ Forms, Google LLC, Mountain View, CA, USA) with the help of a trained research assistant created a ranking questionnaire. The questionnaire consisted of 1l challenges presented by the two groups combined.

The questionnaire was handed to each participant for ranking ideas using a Likert scale of 1–5 scores with 1 representing very low priority and 5 representing highest priority. The ranking process was conducted independently and without discussion. The results were collated and analyzed using a spreadsheet as explained in the data analysis section below.

#### 2.1.8. Data Management

During the nominal group discussions, two types of data were collected; qualitative and quantitative data. Each type of data obtained was first managed individually and then the findings were combined to fully address the main aim of this study. Qualitative data was recorded in chart sheets as well as through a recorder to be analyzed at a later stage. Quantitative data obtained from the ranking tool was entered onto a google spreadsheet for further analysis as explained in the section below. This enabled us to immediately report the results back to the participants and more qualitative data was obtained during clarification to elaborate on the ranking data

### 2.2. Data Analysis

**Figure 2:**
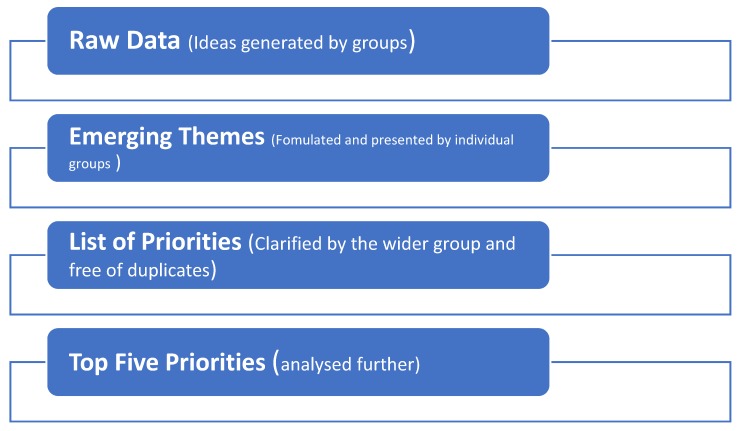
Overview of the NGT Data analysis Process.

Two nominal groups consisting of four participants each were conducted. Data analysis was ongoing and an overview of the process followed is provided in Figure 2. This involved ranking of ideas and thematic analysis of the qualitative data. All stages are described in detail below, where analysis will be discussed in the context of three terms: ideas, priorities and themes [12].

#### 2.2.1. Raw Data Analysis

Ideas raised by participants in the silent brainstorming of each nominal group were presented to the wider group. All the ideas were put up for participants to view simultaneously and the facilitator went on to facilitate grouping of all the ideas according to emerging themes agreed upon by the wider group. Similar ideas were grouped together and duplicates were removed. The themes then became priorities to be voted upon in the ranking stage.

#### 2.2.2. Quantitative Data Analysis

The most common technique to analyze and describe nominal group data is summing the votes allocated to each idea to determine the overall priority score [17,18,19]. This method was employed in this study, where quantitative data obtained from the participants ranking of ideas on a scale of 1–5 was analyzed through the summing of votes allocated to each idea. The overall priority score for each theme was then calculated. This was done through capturing ranking responses into google forms and calculating overall priority scores. A priority list of responses was then drawn and presented to the wider group.

#### 2.2.3. Qualitative Data Analysis

We conducted qualitative analysis of the first five highest overall priority scores. The recorded qualitative data were collected from the participant’s presentations, where the rationale for selecting these themes was provided. More qualitative data collected during the discussion and clarification of the priority list was also utilized to elaborate more on the selected themes.

## 3. Results

### 3.1. Characteristics of Study Participants

In total the NGT team comprised of 8 participants from ages 23–50, with each group comprising of four participants. The attendance rate was 72% since 11 participants were expected. Reasons for nonattendance included other work commitments, inability to organise transportation, other appointments and protest actions on the road. All the stakeholders in attendance reported involvement in POC diagnostics and their specific roles are reported in Table 1.

### 3.2. Nominal Group Ranking

Stakeholders identified a combined total of 18 challenges (Appendix A), which were then categorised into 10 themes. Ranking results of these themes from highest priority to lowest are presented in Table 2.

### 3.3. Thematic Analysis of Top Five Priorities

The study was aimed at determining priority areas for development of a POC diagnostics curriculum for PHC nurses in rural South Africa. From the ten priority areas determined by key stakeholders during the workshop, the top five ranked priorities were: Absence of POC testing curriculum for nurses (90%); absence of training of staff on HIV testing and counselling as lay counsellors are being phased out (86%); absence of Continuous Professional Development (84%); HPIC Tracking and registering patients before testing: Correct Record Keeping (82%) and Staff involvement in POC Management program (80%). Each theme is presented below with supporting quotes.

#### 3.3.1. Absence of POC Testing Curriculum for Nurses

Stakeholders ranked the absence of a curriculum specific to POC diagnostic services for PHC nurses as the highest challenge to be addressed urgently. The main reasons they highlighted were that personnel who performed HIV testing relied on the knowledge they obtained in their past training, each other’s experiences as well as the internet accessed through personal phones. Furthermore, they reported that there were no structured follow-ups to validate what they were doing. Priority is more on getting tests done and meeting targets. When asked about standard operating procedures they responded that these were not available in their clinics and one participant who worked at a clinic that did have one said that SOPs were not readily available and not easily accessible: “not having a standard training is very unfair because we do not even have internet to search when we do not understand something.”

#### 3.3.2. Absence of Training of Staff on HIV Testing and Counselling as Lay Counsellors Are Being Phased Out

Stakeholders reported that they were concerned that no formal training was provided to professional nurses in order to gain skills needed for the provision of quality POC testing, particularly during this period of phasing out of lay counsellors. Participants highlighted that lay councillors were well trained to provide high quality HIV testing and counselling services. In addition, the main duty for lay counsellors was HIV testing, therefore all their attention was on providing this service to the best of their ability: “there is a great need to motivate, encourage and re-train Professional nurses to take over the quality duties of phased out HIV/AIDS lay-councillors, because lay councillors provided more quality testing than professional nurses who have other priorities.”

#### 3.3.3. Absence of Continuous Professional Development

In addition to not having a POC curriculum, stakeholders reported lack of financial support to enrol on additional courses. Stakeholders reported that the shortage of staff in clinics prevented them from securing leave days to attend workshops. They also reported a lack of such opportunities and that they rarely receive invitations to attend workshops. As a result, PHC professionals rely on secondary information from senior nurses who are usually invited to attend workshops: “majority of Professional nurses obtained their qualifications many years ago, having them just be given HIV testing duties on top of their current workload without some form of training is not right”

“From time to time nurses need to be updated or retrained as new technologies change all the time”

#### 3.3.4. HPRS Tracking and Registering Patients before Testing: Correct Record Keeping

Health Patient Registration System (HPRS) is a tracking system that works via the Health Patient Registration Number (HPRN) with a purpose to track and keep a unique record for each patient. Stakeholders ranked the utility of this system for correct record keeping amongst high priority challenges because there are shortages of HPRS registers and there are no other means to support this system to ensure that correct information is captured. Moreover, this system is not linked across different health centres in the country, which then largely affects the reliability of HIV statistics reported for South Africa.

#### 3.3.5. Lack of Staff Involvement in POC Management Programs

Stakeholders who were still on their lay-counsellor duties expressed their disappointment by the lack of interest of professional nurses on POC Management programs. Amongst these programs they highlighted procurement, quality assurance and proficiency testing: “professional nurses always complain about how overworked they are as it is, adding HIV testing on top of all that they have to do is really worrying”; “POC management programs will suffer even more with having no one whose main focus is HIV testing”

## 4. Discussion

This study has identified priority areas for the development of a POC diagnostics curriculum to improve competence and adherence to POC diagnostics quality standards for primary healthcare (PHC) nurses in rural South Africa. From the five highest ranked priorities, four concern the absence of training, Continuing Professional development (CPD) opportunities for PHC professionals and poor staff involvement in HIV programs. The fourth highest ranked priority (HPRS Tracking and registering patients before testing: Correct Record Keeping) is the only priority addressing a different issue. However, this particular priority is a significant and very interesting finding to be looked at further, as it touches issues of record keeping and HIV statistics in the country. The findings of this study also identified gaps in the management and proper implementation of HIV diagnostics in South Africa. This is particularly due to the phasing out of HIV lay counsellors and adding HIV testing to professional nurse duties without a structured training in place to ensure proper transition as well as the sustainability of quality testing services. The findings of this study also demonstrate that identifying of priority areas towards improving the quality of POC diagnostic services has an impact on the achievement of the 90:90:90 goal by 2020, which is a local health priority. In recent years South Africa has made great progress in getting more people tested for HIV; where in 2017 the first of the 90-90-90 targets was met, with 90% of people living with HIV being aware of their status up from 66.2% in 2014 [20,21]. The statistics look impressive, however achieving these targets may lead to increased pressure on nurses performing HIV rapid tests, which could compromise the quality of tests performed as highlighted by the stake holders in this study.

The findings of this study support the wider literature in emphasizing that incompetency of health professionals, lack of CPD, training resources and time constraints remain as barriers to the provision of quality POC services in resource limited settings [3,4,22]. Furthermore, participants in a study conducted in a resource limited setting in India also highlighted high workloads, lack of willingness to participate in POC testing programs, missing support (including training) and pressure to meet targets as contributors to poor quality testing [23]. A report focusing the spotlight on the full achievement of the 90:90:90 goal highlighted healthcare workers attitudes as one of the barriers for patients who want to know their HIV status as well as those who want to return to care [24]. Stakeholder who participated in our study also raised concerns about poor health worker attitudes and lack of staff involvement in HIV management programs as a result of lack of training or too much work pressure, as lay counsellors are being phased out.

In comparison to other research approaches like focus group discussions and in-depth interviews, Stakeholder engagements through the NGT were shown to be effective in identifying priority areas for the development of a POC diagnostics Curriculum. Furthermore, the findings of this study support findings of recent studies conducted in a similar setting [2,3,21,22]. These studies also highlighted lack of support, training opportunities, high workloads and lack of staff involvement as challenges or priority arears to be addressed towards ensuring the provision of quality POC diagnostic services. Stakeholder engagements were further recommended for ensuring effectiveness of future diagnostics as well as for the true potential of POC testing to be realized [4,23].

Strengths of this study include that healthcare users’ views and priorities are increasingly being recognized by policy makers in contributing towards improvement of current policies and practices [12]. A limitation to this study in comparison to other NGT studies may be due to poor availability of stakeholders from four KZN districts, however the participating stakeholders represented a wide variety of relevant role players in the implementation of POC diagnostic services. Moreover, since there were only two groups, all the participants had a fair opportunity to express their views and more time was available for probing and clarifying of ideas. Furthermore, there were no challenges in terms of comparing and presenting larger data sets, which is normally the case for multiple group NGTs [12,25].

Recommendations

Based on the success of the NGT in identifying and ranking priority areas with supporting reasons for the development of a POC curriculum for nurses in rural clinics, we recommend more stakeholder involvement in the development of a context specific POC diagnostic services curriculum for onsite training. This curriculum must be supported by the availability of quality assures in each clinic and regular meetings for staff members to review SOPs and discuss POC diagnostics related issues with management to ensure sustainability.

## 5. Conclusions

This study has presented key stakeholders’ views on the development of a POC diagnostics curriculum to improve competence and adherence to POC diagnostics quality standards for primary healthcare (PHC) nurses in rural South Africa. Phasing out of HIV lay counsellors without a POC curriculum in place, continuous professional development and lack of staff involvement in HIV testing programs were ranked as the highest priority areas that need more focus to ensure delivery of quality POC diagnostic testing at the PHC level. We recommend continuous collaborations between all POC diagnostics stakeholders for the development of an effective and accessible curriculum to ensure quality and sustainable POC diagnostic services in rural PHC clinics.

## Figures and Tables

**Table 1:** Key stakeholder characteristics and involvement in point-of-care (POC) diagnostic services.

District/Institution	Occupation	POC Diagnostics Involvement
Umgungundlovu	Professional Nurse	Testing and HIV Program co-Ordinator
Harry Gwala	Professional Nurse	Full patient consultations, including HIV testing
Uthukela	Professional Nurse	Full patient consultations, including HIV testing
Umzinyathi	TB Professional Nurse	TB and HIV testing
Ilembe	HIV/AIDS lay councillor	HIV Testing
Ethekwini	TB assistant	Previous HIV lay counsellor
University of KZN	Academic Leader	Medical Scientist, Public Health Professor with expertise on implementation research for POC diagnostics
University of KZN	Postdoctoral Fellow	Researcher in implementation and supply chain management of POC diagnostic tests

**Table 2:** Ranking results in descending order.

Item	Summing by Votes Scores					Total%
	5	4	3	2	1	100
No POC Testing Curriculum for nurses	7	2	0	1	0	90
No training of staff on HIV testing and counselling as lay counsellors are being phased out.	7	0	2	1	0	86
Continuous Professional Development	6	1	3	0	0	84
HPIC Tracking and registering patients before testing: Correct Record Keeping	6	2	0	1	1	82
Staff involvement in POC Management program	5	2	2	0	1	80
Pressure on lay councillors due to staff shortage and having to meet targets	4	3	1	1	1	76
Supply Chain Management for POC diagnostics testing	4	3	1	1	1	76
Confidentiality-Stigma	4	2	2	1	1	74
Research Study Process control: Better informed participants to ensure adherence to study timelines	2	1	5	1	1	64
Client Attitude to testing	2	1	5	1	1	64

## Appendix A

Identified list of Priorities for the development of a POC diagnostics Curriculum

1. Continuous Professional Development

2. POC Testing Curriculum for nurses

3. Staff involvement in POC Management program

4. Understaffing

5. Client Attitude to testing

6. Grand mothering phenomenon

7. Consent for testing children

8. Supply Chain Management for POC diagnostics testing

9. Shortage of equipment

10. Shortage of test kits

11. Research Study Process control: Better informed participants to ensure adherence to study timelines

12. Confidentiality

13. Stigma

14. Pressure on lay councilors due to staff shortage and having to meet targets

15. No training of staff on HIV testing and counselling as lay counsellors are being phased out.

16. HPIC Tracking and registering patients before testing: Correct Record Keeping.

17. Incorrect demographic data

18. Non regular System update
